# Association of Preoperative High-Intensity Interval Training With Cardiorespiratory Fitness and Postoperative Outcomes Among Adults Undergoing Major Surgery: A Systematic Review and Meta-Analysis

**DOI:** 10.1001/jamanetworkopen.2023.20527

**Published:** 2023-06-30

**Authors:** Kari Clifford, John C. Woodfield, William Tait, Holly A. Campbell, James Chris Baldi

**Affiliations:** 1 Department of Surgical Sciences, Otago Medical School, University of Otago, Dunedin, New Zealand; 2 Surgical Outcomes Research Centre (SOuRCe), Department of Surgical Sciences, Otago Medical School, University of Otago, Dunedin, New Zealand; 3 Department of Medicine, Otago Medical School, University of Otago, Dunedin, New Zealand

## Abstract

**Importance:**

Preoperative high-intensity interval training (HIIT) is associated with improved cardiorespiratory fitness (CRF) and may improve surgical outcomes.

**Objective:**

To summarize data from studies comparing the association of preoperative HIIT vs standard hospital care with preoperative CRF and postoperative outcomes.

**Data Sources:**

Data sources included Medline, Embase, Cochrane Central Register of Controlled Trials Library, and Scopus databases with no language constraints, including abstracts and articles published before May 2023.

**Study Selection:**

The databases were searched for randomized clinical trials and prospective cohort studies with HIIT protocols in adult patients undergoing major surgery. Thirty-four of 589 screened studies met initial selection criteria.

**Data Extraction and Synthesis:**

A meta-analysis was performed according to Preferred Reporting Items for Systematic Reviews and Meta-analyses (PRISMA) guidelines. Data were extracted by multiple independent observers and pooled in a random-effects model.

**Main Outcomes and Measures:**

The primary outcome was change in CRF, as measured by either peak oxygen consumption (V̇o_2_ peak) or 6-Minute Walk Test (6MWT) distance. Secondary outcomes included postoperative complications; hospital length of stay (LOS); and changes in quality of life, anaerobic threshold, and peak power output.

**Results:**

Twelve eligible studies including 832 patients were identified. Pooled results indicated several positive associations for HIIT when compared with standard care either on CRF (V̇o_2_ peak, 6MWT, anaerobic threshold, or peak power output) or postoperative outcomes (complications, LOS, quality of life), although there was significant heterogeneity in study results. In 8 studies including 627 patients, there was moderate-quality evidence of significant improvement in V̇o_2_ peak (cumulative mean difference, 2.59 mL/kg/min; 95% CI, 1.52-3.65 mL/kg/min; *P* < .001). In 8 studies including 770 patients, there was moderate-quality evidence of a significant reduction in complications (odds ratio, 0.44; 95% CI, 0.32-0.60; *P* < .001). There was no evidence that HIIT differed from standard care in hospital LOS (cumulative mean difference, −3.06 days; 95% CI, −6.41 to 0.29 days; *P* = .07). The analysis showed a high degree of heterogeneity in study outcomes and an overall low risk of bias.

**Conclusions and Relevance:**

The results of this meta-analysis suggest that preoperative HIIT may be beneficial for surgical populations through the improvement of exercise capacity and reduced postoperative complications. These findings support including HIIT in prehabilitation programs before major surgery. The high degree of heterogeneity in both exercise protocols and study results supports the need for further prospective, well-designed studies.

## Key Points

**Question:**

What is the evidence for the efficacy of preoperative high-intensity interval training (HIIT) in improving cardiorespiratory fitness and surgical outcomes?

**Findings:**

This systematic review and meta-analysis of 12 studies including 832 patients assessed the association of preoperative HIIT with either cardiorespiratory fitness or postoperative outcomes. There was evidence that HIIT is significantly associated with increased preoperative cardiorespiratory fitness and reduced postoperative complications.

**Meaning:**

These findings suggest that preoperative HIIT may improve cardiorespiratory fitness and reduce postoperative complications.

## Teaser

This systematic review and meta-analysis summarizes the association of high-intensity interval training (HIIT) with preoperative cardiorespiratory fitness and postoperative complications among adults undergoing major surgery.

## Introduction

Cardiorespiratory fitness (CRF) improves physical and cognitive function and is associated with a lower risk of cardiovascular disease,^1^ diabetes, and cancer^2^; fewer postsurgical complications^3^; and improved health-related quality of life.^4,5^ An expanding body of evidence suggests that CRF can be increased preoperatively, improving postoperative outcomes.^3,6,7^ Surgical recovery increases postoperative oxygen consumption by up to 50%^8,9^ in response to inflammation and to promote tissue healing. Patients who are unable to meet increased oxygen demands because of comorbidities are at a higher risk of complications.^10,11^ Postoperative complications occur in approximately 30% of patients,^12,13^ or up to 50% for frail patients.^14,15^ Most patients are capable of increasing their CRF,^6^ with 1.6 to 2 mL/kg/min considered a clinically and functionally relevant change in peak oxygen consumption (V̇o_2_ peak).^7,16,17^ Frailty is associated with low CRF (V̇o_2_ peak <15 mL/kg/min),^18^ contributed to by physical inactivity, with broad-reaching and detrimental clinical implications.^19^

The limited preoperative time frame requires a targeted approach to increasing CRF. High-intensity interval training (HIIT) is a bolus-dosing approach that efficiently increases CRF^20,21^ and is feasible in most surgical populations.^21^ High-intensity interval training involves repeated aerobic high-intensity intervals at approximately 80% of the maximum heart rate, followed by active recovery. The rapid increases in CRF elicited with HIIT is appealing for preoperative patients, and in the context of pathology, age, and comorbidities, the volume of training stimulus required to improve CRF can often be achieved.^22^

We examined prospective studies comparing HIIT with standard care in patients undergoing major surgery. Previous reviews of prehabilitation are inconclusive or conflicting^23,24^ due to heterogeneous interventions and outcomes impeding the synthesis and interpretation of results or the review being limited to a specific pathology or surgical procedure. Thus, we reviewed the current evidence for preoperative HIIT, including all pathologies and procedures on improving preoperative CRF, and its association with improving postoperative outcomes, including complications, hospital length of stay (LOS), and patient quality of life.

## Methods

This systematic review and meta-analysis of studies comparing prehabilitative HIIT with standard care in patients undergoing major surgery was performed according to Preferred Reporting Items for Systematic Reviews and Meta-analyses (PRISMA) reporting guidelines. The a priori protocol was registered in PROSPERO (CRD42021295341). The review protocol, analysis code, and extracted data are available upon request.

### Eligibility Criteria

We reviewed the use of preoperative HIIT in adult patients, including randomized clinical trials (RCTs) and prospective cohort studies. We included all types of major surgery and all pathologies. Major surgery was defined in individual study methods and could include a procedure expected to last 2 hours or with an anticipated blood loss of greater than 500 mL. Patients performed exercises under supervision in the hospital; in public gyms, local community centers, and physical therapy centers; or unsupervised at home. Exclusion criteria included studies that were not prospective, that did not include HIIT, in which neoadjuvant chemotherapy and/or radiotherapy were administered throughout the exercise intervention, that had an exercise duration longer than 3 months, in which outcomes of interest were not reported,^25^ that had no comparison group, and in which adherence to high-intensity exercise was not achieved in greater than 50% of participants as reported in individual studies.^26^

### Interventions

Included studies examined the outcomes associated with HIIT in presurgical populations. Participants achieved high intensity at approximately 80% maximum heart rate or an equivalent level of intensity according to at least 1 criterion as defined in eTable 1 in Supplement 1. We included studies in which additional interventions other than HIIT were performed at the same time, including education, nutritional and psychological interventions, strength and resistance training, respiratory training, and low-intensity aerobic exercise. Studies in which the comparator groups performed moderate-intensity aerobic training (aerobic exercise that did not meet the intensity targets listed in eTable 1 in Supplement 1) were excluded. Studies in which the exercise group combined both moderate- and high-intensity aerobic training were included.

### Information Sources

PubMed, Embase, Cochrane Central Register of Controlled Trials Library, and Scopus databases and trial registries (ClinicalTrials.gov and the World Health Organization’s International Clinical Trials Registry Platform) were searched, including abstracts and articles published before May 2023. The bibliographies of included studies, clinical practice guidelines, and systematic reviews were hand searched for other relevant articles. There were no language or publication period limitations. The search strategy is included in the eAppendix in Supplement 1.

### Data Extraction

Two researchers (W.T., H.A.C.) independently screened all citations, reviewed abstracts for eligibility, and extracted data, with discrepancies resolved by the senior author (J.C.W.). Data, including study and patient characteristics, intervention details, and outcome measures, were extracted into forms developed from the Cochrane Collaboration’s data extraction template.^27^ Corresponding authors were contacted to clarify information as required. The methodological quality of studies was assessed by 2 reviewers (K.C. and W.T.) using the Cochrane Collaboration’s risk-of-bias tool for RCTs.^28^ Each domain was assessed as high, low, or unclear risk. The quality of evidence was assessed using the Grading of Recommendations Assessment, Development, and Evaluation (GRADE) system.^29^ The therapeutic quality of each study was also assessed using the i-CONTENT tool.^30^

### Data Items

The primary outcome was change in CRF when measured before and after exercise by V̇o_2_ peak or by the 6-Minute Walk Test (6MWT) (the distance in meters walked in 6 minutes). Secondary physiologic outcomes included change in V̇o_2_ at the anaerobic threshold, change in peak power output, and change in endurance time. Secondary clinical outcomes included postoperative complications, which were reported as defined in individual studies and included up to 30 days after surgery. Other clinical postoperative outcomes included hospital LOS, the postoperative morbidity score,^31^ quality of life as recorded in the 36-Item Short Form survey,^32^ mortality within 30 days, and adverse events related to exercise intervention.

Random-effects meta-analysis was performed for these study outcomes using direct comparisons to determine the pooled relative effect of each treatment. When medians (IQR or range) were reported, we estimated means (SDs) as outlined by Wan et al.^33^ When no estimate could be made, SDs were imputed based on the average mean (SD) of other studies for that group (eTable 2 in Supplement 1). Analyses were performed using a frequentist framework in R, version 4.1.2 (R Foundation for Statistical Computing) with the metafor and meta packages. Categorical data were summarized as odds ratios (ORs) and 95% CIs, and continuous data were summarized as cumulative mean differences or standard mean differences if studies used different assessment tools to determine the same outcome. Results were considered significant at *P* ≤ .05 for 2-sided tests. Between-study heterogeneity was evaluated using the *I*^2^ statistic. Sensitivity analyses were planned for the methodological quality of data and level of supervision. A subanalysis was planned for abdominal surgery vs other procedures and to compare outcomes between prehabilitation with HIIT alone and HIIT with other prehabilitation interventions (multimodal HIIT). Publication bias was assessed with funnel plots identifying where studies were possibly missing from the data.^34^

## Results

**Figure 1.**
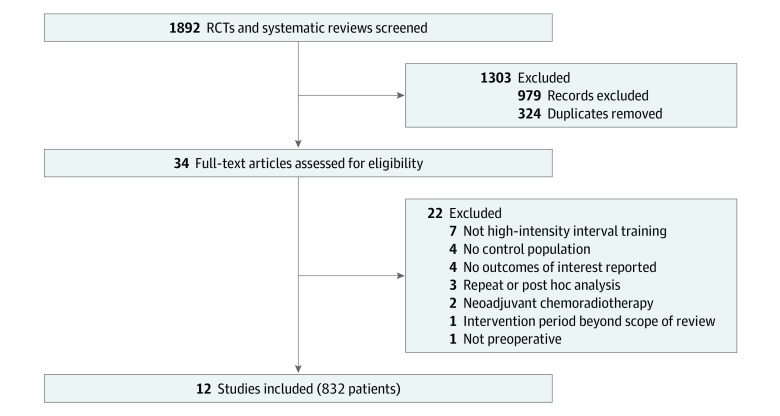
Flow Diagram of Study Inclusion RCT indicates randomized controlled trial.

A total of 354 titles, 235 abstracts, and 34 full-text articles were assessed, from which we identified 12 eligible studies including 832 patients. The PRISMA flowchart is provided in Figure 1. Twenty-two full-text articles were excluded. The most common reasons were that either the study did not investigate HIIT (7 studies), had no control population (4 studies), or did not report on the outcomes of interest (4 studies).

Nine of the studies were RCTs,^3,6,17,35,36,37,38,39,40^ and 3 were observational cohort studies.^16,41,42^ All but 1 study reported the sex of participants.^39^ Of those studies, 181 females (40.2%) and 269 males (59.7%) comprised the intervention groups, and 158 females (34.5%) and 299 males (65.4%) comprised the control groups. The mean (SD) age of participants in included studies was 66.5 (6.1) years for the intervention group and 67.1 (5.9) years in the control group. Other interventions performed at the same time as HIIT were identified in 7 of 12 studies, including counseling and nutritional advice,^3,37,40^ resistance exercise (5 studies),^36,37,40,42,43^ and respiratory training,^39,43^ with 3 studies having 3 interventions.^37,40,43^

The surgical procedures included liver,^17,41^ lung,^39,43^ colorectal,^16,36,37,40,42^ urologic,^35^ and mixed major abdominal .^3,6^ surgeries. In all studies, the comparison group comprised patients undergoing standard care. The studies are summarized in the Table.

**Table.** Characteristics of Included Studies

Source	Design	Participants enrolled, No.		Disease or procedure	Inclusion/exclusion criteria^a^	Outcomes reported	Intervention period, wk	Exercise frequency, per wk	Approximate total intense exercise duration for the intervention period, min	Approximate duration (min) of HIIT + recovery per session	Attendance, % of planned sessions	High-intensity exercise target	Modality
		HIIT	SC										
Banerjee et al,^35^ 2018	RCT	30	30	Radical cystectomy	Included: patients with bladder cancer; excluded: patients requiring urinary diversion	V̇o_2_ peak, complications, LOS	3-6	2	240	30	80	13-15, Borg Rating of Perceived Exertion Scale score (70%-85% HR_max_)	HIIT
Barberan-Garcia et al,^3^ 2018	RCT	73	71	Abdominal surgery	Included: high-risk elective surgery patients; excluded: patients at <4 wk presurgery	Complications, 6MWT, LOS, SF-36	6	2-3	160	40	ND	High = 85% PPO	HIIT + motivational counseling
Berkel et al,^36^ 2022	RCT	39	35	Colorectal resection	Included: MET ≤7, VAT <11	V̇o_2_ peak, complications	3	3	160	60	90	120% of the work rate at VAT	HIIT + resistance training
Dunne et al,^17^ 2016	RCT	20	18	Liver resection	Included: patients with resectable colorectal liver metastases; excluded: chronic liver disease	V̇o_2_ peak, VAT, LOS, complications, SF-36	4	3	Not defined	30	95	>90% V̇o_2_ peak	HIIT
Licker et al,^37^ 2017	RCT	81	83	Lung cancer	Included: NSCLC stage less than IIIA	V̇o_2_ peak, 6MWT, complications	3-4	2-3	80	24	87	80%-100% PPO	HIIT + resistance training + motivational counseling
Molenaar et al,^40^ 2023	RCT	123	128	CRC	Included: patients scheduled for elective CRC surgery; excluded: patients with metastases, ASA >4, chronic kidney failure, or comorbidities contraindicated for exercise	V̇o_2_ peak, 6MWT, complications	4	3	100	24	77	High = 85%-90% PPO	HIIT + resistance training, nutritional, and psychological support
Morkane et al,^41^ 2020	Non-RCT	16	17	Liver transplant	Included: patients with cirrhotic liver disease	V̇o_2_ peak, LOS	6	3	200	18-20	94	Moderate = 80% VAT; high = 50% of difference in work rates between V̇o_2_ peak and VAT	HIIT
Sebio Garcia et al,^43^ 2016	RCT	20	20	Lung cancer	Included: patients with at least 1 of the following: FEV_1_ ≤80% of predicted value, BMI ≥30, aged ≥75 y, or ≥2 comorbidities, and distance to the facility center ≤80 km; excluded: neoadjuvant therapy within 6 mo of enrollment	LOS, SF-36, 6MWT	7-8	3-5	126	30	64	High = 80% PPO	HIIT + resistance training + respiratory training
Stefanelli et al,^39^ 2013	RCT	20	20	Lung cancer	Included: patients aged <75 y, NSCLC stage I-IIA with COPD; excluded: patients with ≥1 of the following: diabetes; CVD; kidney, liver, or respiratory failure; Spo_2_ <90% during the 6MWT	V̇o_2_ peak	3	5	Not defined	Not defined	ND	70% PPO + 10%/wk	HIIT + respiratory training
van Rooijen et al,^42^ 2019	Non-RCT	20	30	CRC	Included: patients with resectable CRC; excluded: patients undergoing chemoradiotherapy, ASA 4-5	Complications, LOS	4	3	Not defined	Not defined	88	High = 85%-100% V̇o_2_ peak	HIIT + resistance training
West et al,^16^ 2015	Non-RCT	22	13	CRC	Included: patients with resectable CRC after neoadjuvant chemoradiotherapy	V̇o_2_ peak	6	3	200	18	96	High = 50% the difference in work rates between V̇o_2_ peak and VAT	HIIT
Woodfield et al,^6^ 2022	RCT	28	35	Abdominal surgery	Included: patients living within the hospital catchment area	V̇o_2_ peak, POMS, LOS, complications, SF-36	4	3	100	20	85	High = 90% max HR	HIIT

Abbreviations: 6MWT, 6-Minute Walk Test; ASA, American Society of Anesthesiology score (classification of physical status); BMI, body mass index (as measured by weight in kilograms divided by height in meters squared); COPD, chronic obstructive pulmonary disease; CRC, colorectal cancer; CVD, cardiovascular disease; FEV_1_, forced expiratory volume in first second of expiration; HIIT, high-intensity interval training; HR_max_, maximum heart rate; LOS, length of hospital stay; MET, metabolic equivalent task; ND, not defined; NSCLC, non–small cell lung cancer; POMS, postoperative morbidity score; PPO, peak power output; RCT, randomized controlled trial; SF-36, 36-Item Short Form; Spo_2_, oxygen saturation as measured by pulse oximetry; VAT, V̇o_2_ at anaerobic threshold (mL/kg/min); V̇o_2_ peak, volume of oxygen used (mL/kg/min).

^a^ All included patients were adults and excluded if unable to participate in exercise training.

The overall quality of evidence is summarized for each outcome according to GRADE guidelines in eTable 3 in Supplement 1. Risk of bias was of some concern for most outcomes. The largest contributor to bias was the lack of masking for participants. Assessor masking was not mentioned in only 4 of the 12 studies.^39,40,41,42^ eFigure 1 in Supplement 1 shows the risk-of-bias scores for each examined category. Therapeutic quality for individual studies is summarized in eTable 4 in Supplement 1. Our criteria for included studies were high-intensity exercise and adequate adherence to the exercise protocol, which, along with the few reported adverse events, led to a high therapeutic quality of included studies.

The number of exercise sessions ranged from 6 to 40, with an estimated median reported duration of intense exercise of 160 minutes (range, 80-240 minutes) (Table). Most included studies explicitly mentioned supervision by trained physiologists or physiotherapists. Three studies did not state that exercise sessions were supervised, but the methods suggested that supervision was likely.^39,41,42^ In 1 study with a supervised and clearly defined exercise program, participants exercised at home.^3^

### End Points Assessing CRF

**Figure 2.**
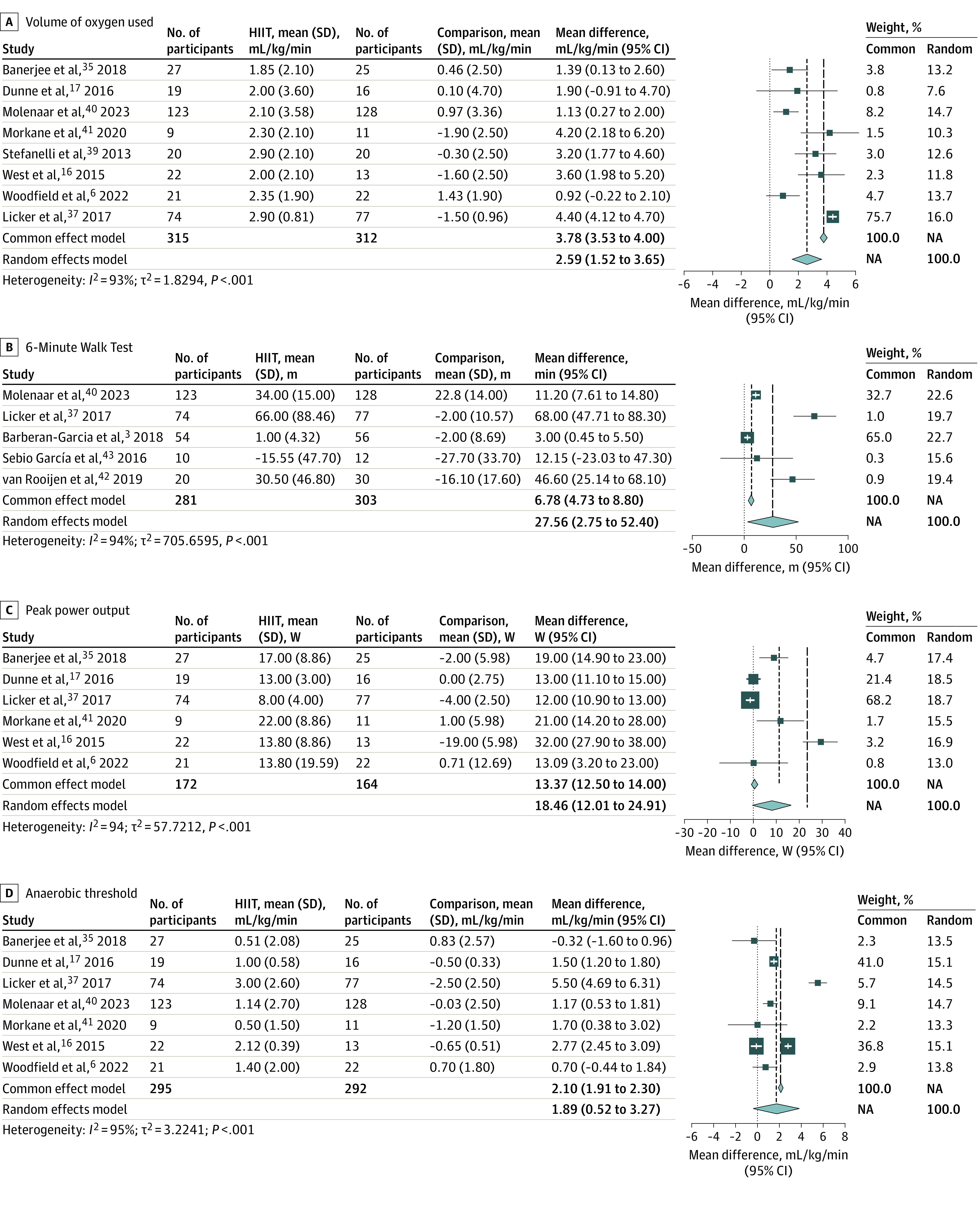
Forest Plots of the Outcomes of High-Intensity Interval Training (HIIT) Compared With Standard Care on Physiologic Outcomes NA indicates not applicable.

Change in V̇o_2_ peak was reported in 8 studies including 627 patients.^6,16,17,35,37,39,40,41^ There was moderate-quality evidence that preoperative HIIT induced a significant improvement in V̇o_2_ peak compared with standard care (cumulative mean difference, 2.59 mL/kg/min; 95% CI, 1.52-3.65 mL/kg/min; *P* < .001). While heterogeneity was significant among study results (*I*^2^ = 93%), in all studies, the mean differences were positive, favoring HIIT (Figure 2A). A funnel plot indicated asymmetry (eFigure 2 in Supplement 1), and a subsequent sensitivity analysis investigating the effect of possible missing studies was performed, finding an estimated 5 missing studies (eFigure 3 in Supplement 1), which would have increased the difference in V̇o_2_ peak if included.

A subanalysis comparing unimodal with multimodal HIIT on a change in V̇o_2_ peak identified 3 multimodal studies using HIIT with separate exercises for resistance and motivational counseling^37,40^ or resistance and pulmonary rehabilitation^39^ and 5 studies reporting V̇o_2_ peak that used only HIIT.^6,16,17,35,41^ Random-effects analysis confirmed that both subgroups had significantly increased V̇o_2_ peak, and this increase did not differ between subgroups (eFigure 4 in Supplement 1). A sensitivity analysis looking at RCTs vs cohort studies for V̇o_2_ peak showed that cohort studies had a slightly larger but nonsignificant mean difference (eFigure 5 in Supplement 1).

The change in the 6MWT was reported in 5 studies including 584 patients.^3,37,40,42,43^ There was low-quality evidence that patients who were enrolled in HIIT walked farther than those in comparison groups (cumulative mean difference, 27.56 m; 95% CI, 2.75-52.40 m; *P* = .03) (Figure 2B). There was significant heterogeneity among study results (*I*^2^ = 94%); however, in all studies, the mean differences favored HIIT. A funnel plot indicated asymmetry in study results (eFigure 6 in Supplement 1).

The change in peak power output was reported in 6 studies including 338 patients.^6,16,17,35,37,41^ There was low-quality evidence that patients enrolled in HIIT reached a higher peak power output on the postintervention cardiopulmonary exercise test (CPET) than those in comparison groups (cumulative mean difference, 18.46 W; 95% CI, 12.01-24.91 W; *P* < .001) (Figure 2C). A funnel plot indicated asymmetry in study results (eFigure 7 in Supplement 1). There was significant heterogeneity between reported results (*I*^2^ = 94%); however, in all studies, the mean differences were greater than 0.

The change in anaerobic threshold was reported in 7 studies including 587 patients.^3,6,16,17,35,37,40,41^ There was very-low-quality evidence that patients who were enrolled in HIIT reached a higher anaerobic threshold on the postintervention CPET than those in comparison groups (cumulative mean difference, 1.89 mL/kg/min; 95% CI, 0.52-3.27 mL/kg/min; *P* = .007) (Figure 2D). There was significant heterogeneity between reported results (*I*^2^ = 95%). A funnel plot indicated asymmetry in study results (eFigure 8 in Supplement 1). Six of the 7 studies reported mean differences greater than 0, favoring HIIT interventions.^3,6,16,17,35,37,40^

### End Points Assessing Postoperative Clinical Outcomes

**Figure 3.**
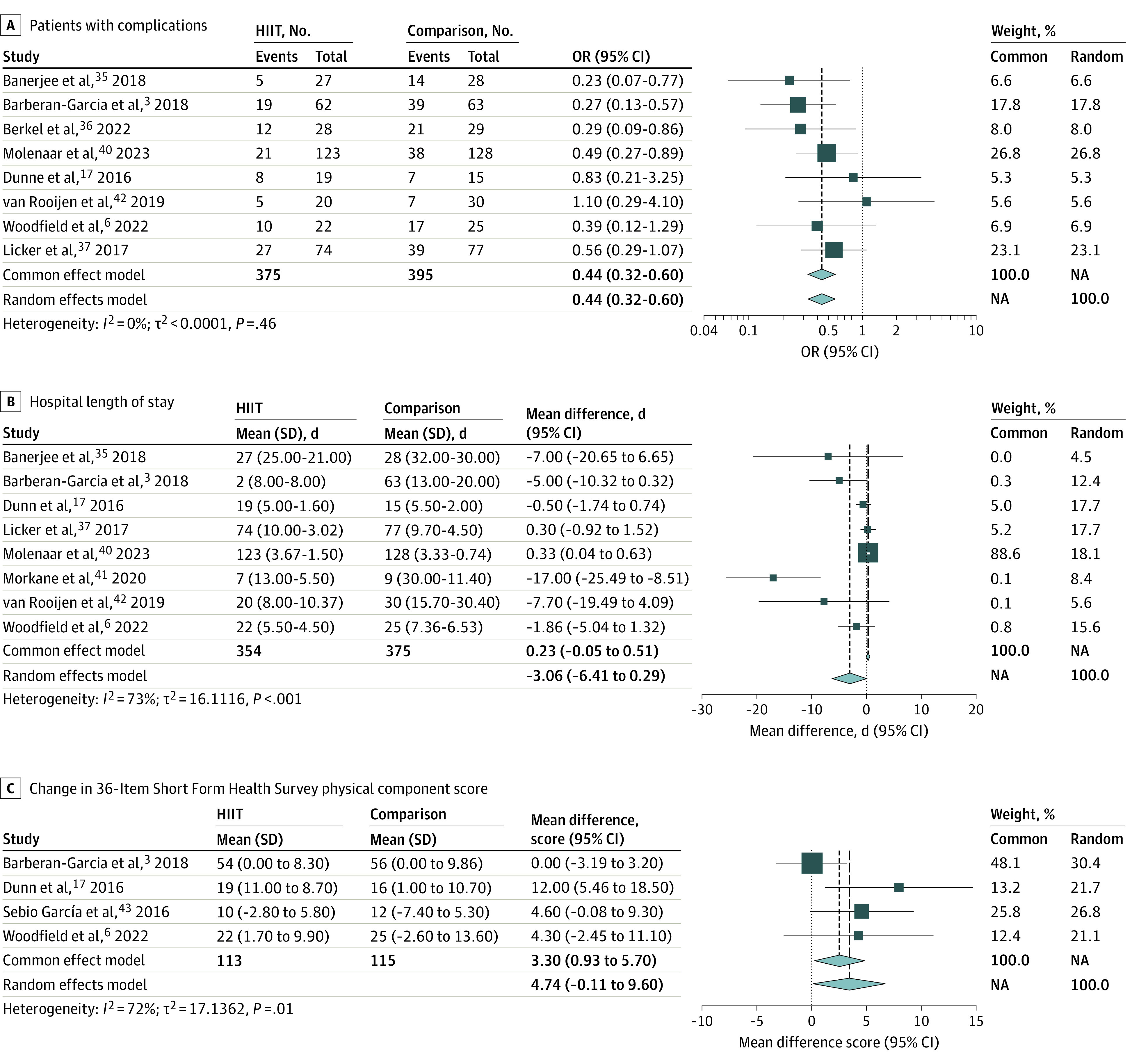
Forest Plots of the Outcomes of High-Intensity Interval Training (HIIT) Compared With Standard Care on Clinical Outcomes and Postoperative Quality of Life NA indicates not applicable.

Eight studies including 770 patients reported the number of patients with postoperative complications.^3,6,17,35,36,37,40,42^ There was moderate evidence that preoperative HIIT reduces the odds of postoperative complications by 56% (OR, 0.44; 95% CI, 0.32-0.60; *P* < .001) (Figure 3A). This effect was also significant when we analyzed data for abdominal surgeries only (OR, 0.45; 95% CI, 0.29-0.68; *P* < .001). There was minimal heterogeneity (*I*^2^ = 0%). There were insufficient data to assess the total number of reported complications. A funnel plot did not suggest asymmetry in study results (eFigure 9 in Supplement 1).

A subanalysis comparing unimodal and multimodal HIIT identified 5 studies using multimodal HIIT, including HIIT plus resistance^36,40,42^ and HIIT plus motivational counseling.^3,37,40^ Three studies used only HIIT.^6,17,35^ Random-effects analysis confirmed that both groups had significantly decreased complications and that the number of patients with postoperative complications did not differ between studies using unimodal or multimodal prehabilitation (OR, 0.40 [95% CI, 0.19-0.82] vs 0.49 [95% CI, 0.32-0.63], respectively; *P* = .63) (eFigure 10 in Supplement 1).

Adverse events were examined by 10 studies including 786 patients.^3,6,16,17,35,37,40,41,42,43^ One study identified a serious adverse event related to HIIT, resulting in participant withdrawal but no clinical sequalae.^6^

Eight studies including 729 patients reported hospital LOS.^3,6,17,35,37,40,41,42^ In 4 studies there was a large decrease,^3,35,41,42^ in 2 a small decrease,^6,17^ and in 2 a small increase^37,40^ in hospital LOS. There was low-quality evidence that HIIT did not differ from standard care in hospital LOS (cumulative mean difference, −3.06 days; 95% CI, −6.41 to 0.29 days; *P* = .07) (Figure 3B). There was significant heterogeneity in individual study results (*I*^2^ = 73%). A funnel plot indicated asymmetry in study results (eFigure 11 in Supplement 1).

Quality of life was assessed using the 36-Item Short Form survey directly after prehabilitation by 4 studies including 214 patients.^3,6,17,43^ There was low-quality evidence that the physical component summary (PCS) score increased significantly after HIIT compared with standard care (cumulative mean difference, 4.74; 95% CI, −0.11 to 9.95; *P* = .06) (Figure 3C), although there was significant heterogeneity (*I*^2^ = 72%). A funnel plot did not suggest asymmetry in study results (eFigure 12 in Supplement 1). One study^6^ assessed PCS 6 weeks after surgery in 47 patients, showing a significant difference in the decrease in score between the HIIT and control groups (−0.82 vs −11.28 points, respectively; *P* = .02). Two studies assessed PCS at 12 weeks after surgery^6,43^ (cumulative mean difference, 6.18 points; 95% CI, −0.57 to 12.9 points; *P* = .07) (eFigure 13 in Supplement 1).

The mental component summary score was reported directly after prehabilitation by 3 studies including 191 patients.^3,6,17^ There was very-low-quality evidence that the change in mental component summary score did not differ significantly between patients participating in HIIT compared with standard care (cumulative mean difference, 2.84 points; 95% CI, −11.20 to 16.89; *P* = .69). There were insufficient data to perform a meta-analysis assessing endurance time at CPET and postoperative morbidity score or to perform a sensitivity analysis assessing the level of supervision and level of adherence to the exercise program.

## Discussion

This systematic review and meta-analysis examined whether a short period of preoperative HIIT with adequate attendance and adherence to intense exercise targets can improve CRF in patients with medical comorbidities. We also documented the association of participation in preoperative HIIT with clinical outcomes.

### Cardiorespiratory Fitness

Preoperative HIIT was associated with an increased V̇o_2_ peak of 2.59 mL/kg/min vs standard care, a result consistent with other reviews.^44,45^ This represents an approximate 10% increase in CRF.

One advantage of HIIT is its ability to rapidly improve CRF. Evidence suggests that approximately 100 minutes of intense exercise can significantly increase CRF.^6,20^ Our results, demonstrating improved CRF with a median time of 160 minutes (range, 80-240 minutes) of intense exercise, are consistent with this hypothesis.

This review is limited by the methodological heterogeneity in exercise interventions, clinical populations, and end point assessments of the included studies. For example, the definitions of high-intensity exercise and protocols (Table) were different in almost every study. While most HIIT programs were less than 4 weeks long, 4 used programs lasting 6 weeks or more,^3,16,38,41^ and additional prehabilitation interventions were inconsistently combined with HIIT. Surgical procedures were in either the abdominal or thoracic cavity and were for a wide range of pathologies. A wide variety of physiologic and clinical end points were used, which may have introduced heterogeneity as studies assess primary end points more carefully than secondary end points.^46^ For example, Berkel et al^36^ reported change in V̇o_2_ peak for the exercise group only, as this was a secondary end point. Despite this heterogeneity, the included studies all showed a clinically relevant improvement in CRF following prehabilitation with HIIT, highlighting the robust efficacy of HIIT and the high therapeutic quality of the included studies.

### Clinical Outcomes

This analysis identified a significant 53% reduction in postoperative complications following HIIT, with minimal heterogeneity. Three prehabilitation RCTs^3,7,40^ powered to assess complications have demonstrated similar results, with an approximate halving of complications. Our meta-analysis supports and strengthens these findings. Previous systematic reviews have not consistently shown an association among prehabilitation, improved CRF, and clinical outcomes^44,46,47,48^ because of heterogeneous protocols and few studies reporting complications. For example, Thomas et al^46^ included 8 studies with 6 moderate and 2 highly intense exercise protocols. Of the 7 studies in their review that reported postoperative outcomes, the only one demonstrating a significant reduction in complications used a HIIT protocol.^3^ In contrast, by including only HIIT studies, our meta-analysis shows a consistent reduction in complications after HIIT programs, even when they were heterogenous. Further study is needed to define optimum HIIT intensities in clinical populations.

Our analysis documented a clinically relevant but nonsignificant reduction in hospital LOS of 3 days, with high heterogeneity among study results. The wide variation in LOS among patients, as well as differences among surgical procedures, resulted in wide CIs. Reviews of prehabilitation showing a reduction in LOS included a study assessing prehabilitation in the context of coronary artery bypass grafts,^49^ and a review by Waterland et al^48^ found that multimodal prehabilitation reduced LOS, although LOS was only reported in 4 studies. Larger studies are required to assess whether HIIT reduces LOS, a result that would be of interest to health administrators. The cost of HIIT was reported in 1 European study^49^ that identified cost savings compared with standard care related to reductions in complications and readmissions. The reduction in complications and LOS in this meta-analysis suggests that supervised preoperative HIIT may be cost-effective, although further research is required to confirm this finding.

While improvement in the PCS quality-of-life score after HIIT did not reach significance, there was a difference noted 6 weeks after surgery. As the PCS measures physical activity and roles, the improvement after HIIT is related to an improvement in CRF, and differences in PCS shortly after surgery may be a good measurement of postoperative recovery.^11^ Further measurement of quality of life after HIIT will be helpful in assessing the benefits of exercise on physical and mental health.

### Limitations

Limitations of this review include study heterogeneity, incomplete reporting of results, limited sample size (total number of participants, 832), and lack of masking. Challenges with methodological heterogeneity of the included studies have been discussed. Statistical heterogeneity was due to a need for additional calculations^37^ based on the data as described in the Methods and summarized in the eAppendix in Supplement 1. While participant masking is an unavoidable limitation in prehabilitation studies, as participants cannot be masked to their exercise intervention, assessor masking was not mentioned in 4 of the 12 studies,^39,40,41,42^ potentially limiting the assessment of clinical outcomes.^50^ For these reasons, when using GRADE, we determined the certainty of the evidence to be moderate (for V̇o_2_ peak and complications) to very low for our outcomes (eTable 4 in Supplement 1). Other limitations of this study include the inability to stratify the population based on patient frailty and the limited data on HIIT in orthopedic patients. Further research is needed in these areas. An advantage of our meta-analysis is that focusing solely on HIIT has enabled its role in prehabilitation to be quantified.

## Conclusions

In this systematic review and meta-analysis, pooled results indicated several positive associations of HIIT vs standard care with CRF or postsurgical outcomes. These findings suggest that HIIT may improve patient outcomes, with robust benefits across patient populations. Preoperative HIIT shows promising results and should be included in prehabilitation programs. The high degree of heterogeneity in our analysis demonstrates differences in training programs and supports the need for further well-designed studies to improve the quality of evidence and confirm effective HIIT protocols.

## Notes

Supplement 1. **eAppendix.** Search Strategy **eTable 1.** Accepted Criteria for Defining Moderate- and High-Intensity Interval Aerobic Exercise **eTable 2.** Data for Noncategorical Variables (Notation Indicates Transformation or Imputation) **eFigure 1.** Risk-of-Bias Plot for Each Outcome and Study **eTable 3.** GRADE Summary of Quality of Evidence **eTable 4.** i-CONTENT Results for Included Studies’ Therapeutic Quality **eFigure 2.** Funnel Plot for Meta-analysis of Change in V̇O_2_ Peak in Included Studies **eFigure 3.** Funnel Plot for Pooled Analysis of Studies Comparing HIIT With Standard Care on V̇O_2_ Peak With Included Missing Studies **eFigure 4.** Forest Plot for Change in V̇O_2_ Peak Subgrouped by Number of Prehabilitation Interventions Included in Individual Studies **eFigure 5.** Forest Plot Showing the Mean Difference Between HIIT and Control Groups, Stratified by RCT and Cohort Study Results **eFigure 6.** Funnel Plot for Meta-analysis of Change in 6-Minute Walk Test in Included Studies **eFigure 7.** Funnel Plot for Meta-analysis of Change in Peak Power Output in Included Studies **eFigure 8.** Funnel Plot for Meta-analysis of Change in Anaerobic Threshold in Included Studies **eFigure 9.** Funnel Plot for Meta-analysis of Number of Patients With Complications in Included Studies **eFigure 10.** Forest Plot for Number of Patients With Complications Subgrouped by Number of Prehabilitation Interventions Included in Individual Studies **eFigure 11.** Funnel Plot for Meta-analysis of Length of Stay in Included Studies **eFigure 12.** Funnel Plot for Change in Physical Component Scores of the SF-36 Quality of Life Questionnaire **eFigure 13.** Pooled Analysis for Studies Assessing the Physical Component Score of the SF-36 Quality of Life Questionnaire at Baseline and at 3 Months Postsurgery (Sebio-García et al) and 12 Weeks (Woodfield et al)

Supplement 2.
Data Sharing Statement
