# Errors in Figure 3 and Supplement

**DOI:** 10.1001/jamanetworkopen.2023.27575

**Published:** 2023-07-19

In the Original Investigation titled “Association of Preoperative High-Intensity Interval Training With Cardiorespiratory Fitness and Postoperative Outcomes Among Adults Undergoing Major Surgery: A Systematic Review and Meta-Analysis,”^1^ published June 30, 2023, in Figure 3 panels B and C, the columns of data for the HIIT and comparison groups, which present the total patient numbers followed by means and SDs, had been incorrectly presented as means with ranges; additionally, a row of data was missing in eTable 2 in Supplement 2. This article has been corrected.^1^
