# Supplementary material for: Association of Preoperative High-Intensity Interval Training With Cardiorespiratory Fitness and Postoperative Outcomes Among Adults Undergoing Major Surgery: A Systematic Review and Meta-Analysis
Source: JAMA Netw Open. 2023 Jun 30;6(6):e2320527. doi: 10.1001/jamanetworkopen.2023.20527 (PMC10314310; doi:10.1001/jamanetworkopen.2023.20527)
Supplement: Supplement 1. — eAppendix. Search Strategy eTable 1. Accepted Criteria for Defining Moderate- and High-Intensity Interval Aerobic Exercise eTable 2. Data for Noncategorical Variables (Notation Indicates Transformation or Imputation) eFigure 1. Risk-of-Bias Plot for Each Outcome and Study eTable 3. GRADE Summary of Quality of Evidence eTable 4. i-CONTENT Results for Included Studies’ Therapeutic Quality eFigure 2. Funnel Plot for Meta-analysis of Change in V̇O2 Peak in Included Studies eFigure 3. Funnel Plot for Pooled Analysis of Studies Comparing HIIT With Standard Care on V̇O2 Peak With Included Missing Studies eFigure 4. Forest Plot for Change in V̇O2 Peak Subgrouped by Number of Prehabilitation Interventions Included in Individual Studies eFigure 5. Forest Plot Showing the Mean Difference Between HIIT and Control Groups, Stratified by RCT and Cohort Study Results eFigure 6. Funnel Plot for Meta-analysis of Change in 6-Minute Walk Test in Included Studies eFigure 7. Funnel Plot for Meta-analysis of Change in Peak Power Output in Included Studies eFigure 8. Funnel Plot for Meta-analysis of Change in Anaerobic Threshold in Included Studies eFigure 9. Funnel Plot for Meta-analysis of Number of Patients With Complications in Included Studies eFigure 10. Forest Plot for Number of Patients With Complications Subgrouped by Number of Prehabilitation Interventions Included in Individual Studies eFigure 11. Funnel Plot for Meta-analysis of Length of Stay in Included Studies eFigure 12. Funnel Plot for Change in Physical Component Scores of the SF-36 Quality of Life Questionnaire eFigure 13. Pooled Analysis for Studies Assessing the Physical Component Score of the SF-36 Quality of Life Questionnaire at Baseline and at 3 Months Postsurgery (Sebio-García et al) and 12 Weeks (Woodfield et al) [file jamanetwopen-e2320527-s001.pdf]

Supplemental Online Content

Clifford K, Woodfield JC, Tait W, Campbell HA, Baldi JC. Association of preoperative high-intensity interval training with cardiorespiratory fitness and postoperative outcomes among adults undergoing major surgery: a systematic review and meta-analysis. *JAMA Netw Open*. 2023;6(6):e2320527. doi:10.1001/jamanetworkopen.2023.20527

eAppendix. Search Strategy

eTable 1. Accepted Criteria for Defining Moderate- and High-Intensity Interval Aerobic Exercise

eTable 2. Data for Noncategorical Variables (Notation Indicates Transformation or Imputation)

eFigure 1. Risk-of-Bias Plot for Each Outcome and Study

eTable 3. GRADE Summary of Quality of Evidence

eTable 4. i-CONTENT Results for Included Studies' Therapeutic Quality

eFigure 2. Funnel Plot for Meta-analysis of Change in $\dot{V}O_2$ Peak in Included Studies

eFigure 3. Funnel Plot for Pooled Analysis of Studies Comparing HIIT With Standard Care on $\dot{V}O_2$ Peak With Included Missing Studies

eFigure 4. Forest Plot for Change in $\dot{V}O_2$ Peak Subgrouped by Number of Prehabilitation Interventions Included in Individual Studies

eFigure 5. Forest Plot Showing the Mean Difference Between HIIT and Control Groups, Stratified by RCT and Cohort Study Results

eFigure 6. Funnel Plot for Meta-analysis of Change in 6-Minute Walk Test in Included Studies

eFigure 7. Funnel Plot for Meta-analysis of Change in Peak Power Output in Included Studies

eFigure 8. Funnel Plot for Meta-analysis of Change in Anaerobic Threshold in Included Studies

eFigure 9. Funnel Plot for Meta-analysis of Number of Patients With Complications in Included Studies

eFigure 10. Forest Plot for Number of Patients With Complications Subgrouped by Number of Prehabilitation Interventions Included in Individual Studies

eFigure 11. Funnel Plot for Meta-analysis of Length of Stay in Included Studies

eFigure 12. Funnel Plot for Change in Physical Component Scores of the SF-36 Quality of Life Questionnaire

eFigure 13. Pooled Analysis for Studies Assessing the Physical Component Score of the SF-36 Quality of Life Questionnaire at Baseline and at 3 Months Postsurgery (Sebio-García et al) and 12 Weeks (Woodfield et al)

This supplemental material has been provided by the authors to give readers additional information about their work.

© 2023 Clifford K et al. *JAMA Network Open*.

eAppendix. Search Strategy

Ovid Embase
exp case control study/ OR exp cohort analysis/ OR exp randomized controlled trial/ OR exp intervention study/ OR exp prospective study/ OR exp controlled study/ OR exp observational study/ OR exp pilot study/ OR exp feasibility study
OR (Case Control OR Cohort Stud* OR Randomi#ed Control* Trial OR Randomi#ed Clinical Trial OR RCT OR Interventional Study OR Prospective Stud* OR Control Study OR Observational Stud* OR Pilot Project* OR Feasibility Study OR Feasibility Trial OR Pilot Stud*).mp.
AND
exp preoperative period/
OR (Preoperative OR Pre-operative OR Pre-operational OR Preoperational OR Preop OR Pre-surgical OR Pre-surgery OR Pre-treatment OR Pre Surgical Intervention OR Periop OR Perioperational OR Perioperative OR Surgical OR Operational OR Surgery OR Operation).mp.
AND
(Prehabilitation OR Prehabilitative OR Prehab OR Preoperative Rehabilitation OR Preoperative Intervention OR Preoperative Care OR Preoperative Training OR Preoperative Exercise OR Preoperative Treatment OR Preoperative Therapy OR Preoperative Exercise Therapy).mp.
AND
exp postoperative complication/ OR exp quality of life/
OR (Postoperative Outcomes OR Postoperative Recovery OR Postoperative Complications OR Surgical Complications OR Postoperative Morbidity OR Postoperative Mortality OR Postoperative Period OR Adverse Events OR Adverse Outcomes OR Quality of Life OR Postoperative Morbi-mortality OR Postoperative Morbimortality OR Postop QoL OR QoL).mp.
AND
exp oxygen consumption/ OR exp endurance/ OR exp exercise test/ OR exp six minute walk test/
OR (VO2 peak OR VO2peak OR Peak VO2 OR VO2 max OR VO2max OR Maximal Oxygen Uptake OR Peak Oxygen Uptake OR Anaerobic Threshold OR Lactate Threshold OR Lactic Acid Threshold OR VO2AT OR VO2 AT OR Peak Exercise Capacity OR Peak Oxygen Consumption OR Peak Physical Endurance OR Physical Endurance OR Cardiopulmonary Exercise Test OR CPET OR CPEX OR Exercise Test OR Cardiopulmonary Exercise Testing OR Six-Minute Walk Test OR Six Minute Walk Test OR Six-Minute Walk Distance OR Six Minute Walk Distance OR 6MWD OR 6MWT OR 6-MWT OR 6MWD OR 6-MWD).mp.

PubMed
((((((((((((((((((Case Control) OR Case-Control) OR Cohort Study) OR Cohort Studies) OR Prospective Cohort Studies) OR Prospective Cohort Study) OR ((((((((((((((((Randomised Control Trial) OR Randomised Controlled Trial) OR Randomised Clinical Trial) OR RCT) OR Interventional Study) OR Prospective Studies) OR Prospective Study) OR Controlled Study) OR Control Study) OR Observational Study) OR Observational Studies) OR Pilot Projects) OR Feasibility Study) OR Feasibility Trial) OR Pilot Study) OR Pilot Studies))) OR Randomized Controlled Study) OR Randomized Controlled Trial) OR Randomized Control Study) OR Randomized Control Trial)))
AND

© 2023 Clifford K et al. *JAMA Network Open*.

(((((((((((((((((Preoperative) OR Pre-operative) OR Pre-operational) OR Preop) OR Preoperational) OR Pre-surgical) OR Pre-surgery) OR Pre-treatment) OR Pre Surgical Intervention) OR Periop) OR Perioperational) OR Perioperative) OR Surgical) OR Operational) OR Surgery) OR Operation)))
AND
(((((((((Prehabilitation) OR Prehabilitative) OR Prehab) OR Preoperative Rehabilitation) OR Preoperative Intervention) OR Preoperative Care) OR Preoperative Training) OR Preoperative Exercise) OR Preoperative Treatment) OR Preoperative Therapy) OR Preoperative Exercise Therapy))))
AND
((((((((((((((((((((((((((((((((Postoperative Outcomes) OR Postoperative Recovery) OR Postoperative Complications) OR Surgical Complications) OR Postoperative Morbidity) OR Postoperative Mortality) OR Postoperative Period) OR Adverse Events) OR Adverse Outcomes) OR Quality of Life))) OR Postoperative Morbi-mortality) OR Postoperative Morbimortality) OR Postop QoL) OR QoL) OR Treatment Outcome))
AND
((VO2 peak) OR VO2peak) OR Peak VO2) OR VO2 max) OR VO2max) OR Maximal Oxygen Uptake) OR Peak Oxygen Uptake) OR Anaerobic Threshold) OR Lactate Threshold) OR Lactic Acid Threshold) OR VO2AT) OR VO2 AT) OR Peak Exercise Capacity) OR Peak Oxygen Consumption)))))))) OR Peak Physical Endurance) OR Physical Endurance) OR Cardiopulmonary Exercise Test) OR CPET) OR CPEX) OR Exercise Test) OR Cardiopulmonary Exercise Testing) OR Ventilatory Threshold)))))))))))))))))))) OR ((((((((((((((((((((((((((((((((Six-Minute Walk Test) OR Six Minute Walk Test) OR Six-Minute Walk Distance) OR Six Minute Walk Distance) OR 6MWD) OR 6MWT) OR 6-MWT) OR 6MWD) OR 6-MWD)))))))))))))))))))))))))))))))))))

Cochrane

"Case Control" OR "Case-Control" OR "Cohort Study" OR "Randomised Controlled Trial" OR "Randomised Clinical Trial" OR rct OR "Interventional Study" OR "Prospective Study" OR "Controlled Study" OR "Observational Study" OR "Pilot Project" OR "Feasibility Study" OR "Feasibility Trial"
AND
"Preop*" OR "Pre-op*" OR "Pre-surg*" OR pre-treatment OR "Pre Surgical Intervention" OR periop* OR surg* OR operation*
AND
prehab* OR "Preop* Rehab*" OR "Preop* Intervention" OR "Preoperative Care" OR "Preoperative Training" OR "Preoperative Exercise" OR "Preoperative Treatment" OR "Preoperative Therapy" OR "Preoperative Exercise Therapy"
AND
"Postoperative Outcome*" OR "Postoperative Recovery" OR "Postoperative Complication*" OR "Surgical Complications" OR "Postoperative Morbidity" OR "Postoperative Mortality" OR "Postoperative Period" OR "Adverse Events" OR "Adverse Outcomes" OR "Quality of Life" OR "Postoperative Morbi-mortality" OR "Postoperative Morbimortality" OR "Postop* QoL" OR qol OR "Treatment Outcome"
AND "VO2 peak" OR vo2peak OR "Peak VO2" OR "VO2 max" OR "VO2max" OR "Maximal Oxygen Uptake" OR "Peak Oxygen Uptake" OR "Anaerobic Threshold" OR "Lactate Threshold" OR "Lactic Acid Threshold" OR vo2at OR "VO2 AT" OR "Peak

© 2023 Clifford K et al. *JAMA Network Open*.

Exercise Capacity" OR "Peak Oxygen Consumption" OR "Peak Physical Endurance" OR "Physical Endurance" OR "Cardiopulmonary Exercise Test*" OR cpet OR cpex OR "Exercise Test" OR "Ventilatory Threshold" OR "Six-Minute Walk Test" OR "Six Minute Walk Test" OR "Six-Minute Walk Distance" OR "Six Minute Walk Distance"

Scopus

TITLE-ABS-KEY (“Case Control” OR “Case-Control” OR “Cohort Study” OR “Randomised Controlled Trial” OR “Randomised Clinical Trial” OR RCT OR “Interventional Study” OR “Prospective Study”
OR
“Controlled Study” OR “Observational Study” OR “Pilot Project” OR “Feasibility Study” OR “Feasibility Trial”
AND
Preoperative OR Pre-operative OR Preoperational OR Pre-operational OR Preop OR Pre-surg* OR Pre-treatment OR “Pre Surgical Intervention” OR Periop OR Perioperational OR Perioperative OR Surgical OR Operational OR Surgery OR Operation
AND
Prehabilitation OR Prehab OR “Preoperative Rehabilitation” OR “Preoperative Intervention” OR “Preoperative Care” OR “Preoperative Training” OR “Preoperative Exercise” OR “Preoperative Treatment” OR “Preoperative Therapy” OR “Preoperative Exercise Therapy”
AND
“Postoperative Outcomes” OR “Postoperative Recovery” OR “Postoperative Complications” OR “Surgical Complications” OR “Postoperative Morbidity” OR “Postoperative Mortality” OR “Postoperative Period”
OR
“Adverse Events” OR “Adverse Outcomes” OR “Quality of Life” OR “Postoperative Morbi-mortality” OR “Postoperative Morbimortality” OR “Postop QoL” OR QoL
AND
“VO2 peak” OR VO2peak OR “Peak VO2” OR “VO2 max” OR VO2max OR “Maximal Oxygen Uptake” OR “Peak Oxygen Uptake” OR “Anaerobic Threshold” OR “Lactate Threshold” OR “Lactic Acid Threshold”
OR
VO2AT OR “VO2 AT” OR “Peak Exercise Capacity” OR “Peak Oxygen Consumption” OR “Peak Physical Endurance” OR “Physical Endurance” OR “Cardiopulmonary Exercise Test” OR CPET OR CPEX OR “Exercise Test”
OR
“Cardiopulmonary Exercise Testing” OR “Six-Minute Walk Test” OR “Six Minute Walk Test” OR “Six-Minute Walk Distance” OR “Six Minute Walk Distance” OR 6MWD OR 6MWT OR 6-MWT OR 6-MWD)

© 2023 Clifford K et al. *JAMA Network Open*.

eTable 1. Accepted Criteria for Defining Moderate and High-Intensity Interval Aerobic Exercise

Criteria	Moderate Intensity	High Intensity Target*
Heart rate	>50% and <80% HR_{peak}	>80% HR_{peak}
Heart rate reserve	>40% <65% HRR	>65% HRR
Oxygen consumption	>40% and <60% $\dot{V}O_{2peak}$	>60% $\dot{V}O_{2peak}$
Power output	>50% and <80% PPO	>80% PPO
Revolutions per minute	>50 and <80 RPM	>80 RPM
Metabolic equivalent	>3 and <6 METS	>6 METS
Borg scale 0-10	>3 and <7.5	>7.5
Borg scale 6-20	>11 and <15	>15

**Peak as determined on Cardiopulmonary Exercise Testing. HR: Heart Rate, HRR: Heart rate reserve, $\dot{V}O_{2peak}$: PPO: Peak power output*

Summary of statistical imputation and transformation

eTable 2. Data for Noncategorical Variables (Notation Indicates Transformation or Imputation)

	HIIT			Standard Care		
**Study**	**Number**	**Mean**	**Standard deviation**	**Number**	**Mean**	**Standard deviation**
**Change in $\dot{V}O_{2peak}$ (mL·kg^{-1}·min^{-1})**						
Banerjee 2018 [34] †	27	1.85	2.1	25	0.46	2.5
Dunne 2016 [17] ‡	19	2	3.6	16	0.1	4.7
Licker 2017 [36]*	74	2.9	0.81	77	-1.5	0.96
Morkane 2020 [40] †	9	2.3	2.1	11	-1.9	2.5
Stefanelli 2013 [39]†	20	2.9	2.1	20	-0.3	2.5
West 2015 [16]SS	22	2	2.1	13	-1.6	2.5
Woodfield 2022 [6]	21	2.35	1.9	22	1.43	1.9
**Change in 6-minute walk test (M)**						
Barberan-Garcia 2018 [5]†	54	1	4.32	56	-2	8.69
Licker 2017 [36]*	74	66	88.46	77	-2	10.57
Molenaar 2023 [39]*	123	34	15.0	128	22.8	14.0
Sebio Garcia 2016 [42]†	10	-15.55	47.7	12	-27.7	33.7
Van Rooijen 2019 [41] †	20	30.5	46.8	30	-16.1	17.6
**Change in Peak Power Output (W)**						
Banerjee 2018 [34] †	27	17	8.86	25	-2	5.98
Dunne 2016 [17] ‡	19	13	3	16	0	2.75
Licker 2017 [36]*	74	8	4	77	-4	2.5

© 2023 Clifford K et al. *JAMA Network Open*.

Study	HIIT Number	HIIT Mean	HIIT Standard deviation	Standard Care Number	Standard Care Mean	Standard Care Standard deviation
Morkane 2020 [40] †	9	22	8.86	11	1	5.98
West 2015 [16]*SS	22	13.8	8.86	13	-19	5.98
Woodfield 2022 [6]	21	13.8	19.59	22	0.71	12.69
**Change in $\dot{V}O_2$ at Anaerobic Threshold (mL·kg^{-1}·min^{-1})**						
Banerjee 2018 [34] †	30	0.51	2.08	30	0.83	2.57
Dunne 2016 [17] ‡	19	1	0.575	16	-0.5	0.325
Licker 2017 [36]*	74	3	2.6	77	-2.5	2.5
Molenaar 2023[39]*	123	1.14	2.7	128	-0.03	2.5
Morkane 2020 [40] †	9	-0.4	2.6	11	-0.4	3.5
West 2015 [16]SS	22	2.12	0.39	13	-0.65	0.507
Woodfield 2022 [6]	21	1.4	2	22	0.7	1.8
**Length of hospital stay (days)**						
Banerjee 2018 [34] P	27	25	21	28	32	30
Barberan-Garcia 2018 [5]†	62	8	8	63	13	20
Dunne 2016 [17] *	19	5	1.6	15	5.5	2
Licker 2017 [36]*	74	10	3.02	77	9.7	4.5
Molenaar 2023 [39]*	123	3.6	1.5	128	3.3	0.75
Morkane 2020 [40] *	7	13	5.5	9	30	11.4
Van Rooijen 2019 [41]*	20	8	10.37	30	15.7	30.4
Woodfield 2022 [6]	22	5.5	4.5	25	7.36	6.53
**Change in the Physical Component Score of the SF-36 Quality of Life Questionnaire**						
Barberan-Garcia 2018 [5]†	54	0	8.31	56	0	9.86
Dunne 2016 [17] ‡	19	13	8.7	16	1	10.7
Sebio Garcia 2016 [42]	10	-2.8	5.8	12	-7.4	5.3
Woodfield 2022 [6]	22	1.7	9.9	25	-2.6	13.6

* Mean was estimated from median (IQR)

† Standard Deviation of the change from baseline to post intervention was imputed

‡ Standard Deviation of the change from baseline to post intervention was calculated from 95% CI

SS Change in variable from week 0 to week 3 (post intervention), Standard Deviation of the change from baseline to post intervention was imputed

P Mean was estimated from median (range)

© 2023 Clifford K et al. *JAMA Network Open*.

eFigure 1. Risk of Bias Plot for Each Outcome and Study

Study ID	Experimental	Comparator	Outcome	Weight	D1	D2	D3	D4	D5	Overall
Licker [37]	HIIT	SC	6MWT	1	+	!	+	!	!	!
Molenaar [40]	HIIT	SC	6MWT	1	+	!	+	+	+	!
Barberan-Garcia [5]	HIIT	SC	6MWT	1	+	+	+	!	+	!
Sebio-Garcia [43]	HIIT	SC	6MWT	1	!	+	+	+	!	!
VanRooijen [42]	HIIT	SC	6MWT	1	!	+	+	+	!	!
Banerjee [35]	HIIT	SC	AT	1	!	+	+	+	!	!
Dunne [17]	HIIT	SC	AT	1	!	+	+	+	!	!
Licker [37]	HIIT	SC	AT	1	+	+	+	!	!	!
Morkane [41]	HIIT	SC	AT	1	-	-	+	+	!	-
West [16]	HIIT	SC	AT	1	-	+	+	+	+	-
Woodfield [6]	HIIT	SC	AT	1	+	+	+	+	+	+
Berkel [36]	HIIT	SC	Complications	1	!	+	+	+	+	!
Banerjee [34]	HIIT	SC	Complications	1	!	+	+	+	!	!
Licker [37]	HIIT	SC	Complications	1	+	+	+	!	!	!
Dunne [17]	HIIT	SC	Complications	1	!	+	+	+	!	!
Molenaar [40]	HIIT	SC	Complications	1	+	!	+	+	+	!
Woodfield [6]	HIIT	SC	Complications	1	+	+	+	+	+	+
Banerjee [35]	HIIT	SC	LOS	1	!	+	+	+	!	!
Dunne [17]	HIIT	SC	LOS	1	!	+	+	+	!	!
Licker [37]	HIIT	SC	LOS	1	+	+	+	!	!	!
Morkane [41]	HIIT	SC	LOS	1	-	-	+	+	!	-
Barberan-Garcia [5]	HIIT	SC	LOS	1	+	+	+	+	+	+
Van Rooijen [42]	HIIT	SC	LOS	1	!	+	+	+	!	!
Dunne [17]	HIIT	SC	PCS	1	!	+	+	+	!	!
Sebio-Garcia [43]	HIIT	SC	PCS	1	!	+	+	+	!	!
Woodfield [6]	HIIT	SC	PCS	1	+	+	+	+	+	+
Banerjee [35]	HIIT	SC	PPO	1	!	+	+	+	!	!
Dunne [17]	HIIT	SC	PPO	1	!	+	+	+	!	!
Licker [36]	HIIT	SC	PPO	1	+	!	+	!	!	!
Morkane [41]	HIIT	SC	PPO	1	-	-	+	+	!	-
West [16]	HIIT	SC	PPO	1	-	+	+	+	+	-
Woodfield [6]	HIIT	SC	PPO	1	+	+	+	+	+	+
Banerjee [35]	HIIT	SC	V O2peak	1	!	+	+	+	!	!
Dunne [17]	HIIT	SC	VO2peak	1	!	+	+	+	!	-
Licker [37]	HIIT	SC	VO2peak	1	+	!	+	!	!	!
Morkane [41]	HIIT	SC	VO2peak	1	-	-	+	+	!	-
Stefanelli [39]	HIIT	SC	VO2peak	1	!	+	+	+	!	!
West [16]	HIIT	SC	VO2peak	1	-	+	+	+	+	-
Woodfield [6]	HIIT	SC	VO2peak	1	+	+	+	+	+	+

\+ Low risk
! Some concerns
\- High risk

D1 Randomisation process
D2 Deviations from the intended interventions
D3 Missing outcome data
D4 Measurement of the outcome
D5 Selection of the reported result

$\dot{V}O_{2peak}$: volume of oxygen used, given as mL/kg^{-1}/min^{-1}
LOS: Length of hospital stay
PPO: Peak Power Output
6MWT: Six minute walk test
AT: $\dot{V}O_2$ at anaerobic threshold, given as mL/kg^{-1}/min^{-1}

© 2023 Clifford K et al. *JAMA Network Open*.

eTable 3. GRADE Summary of Quality of Evidence

Outcome	Risk of Bias	Inconsistency	Indirectness	Imprecision	Publication Bias	Overall Quality of Evidence
Change in $\dot{V}O_{2peak}$	Moderate	High	Low	Moderate	Low	⊕⊕⊕O
Change in 6 minute walk test	Moderate	High	Low	High	Low	⊕⊕OO
Change in peak power output	Moderate	High	Low	High	Low	⊕⊕OO
Change in anaerobic threshold	Moderate	High	High	High	Low	⊕OOO
Postoperative complications	Moderate	Low	Low	Moderate	Low	⊕⊕⊕O
Length of stay	Moderate	Moderate	Low	High	Low	⊕⊕OO
Quality of life (PCS)	Moderate	High	Low	High	Low	⊕⊕OO
Quality of life (MCS)	High	High	High	High	High	⊕OOO

High	⊕⊕⊕⊕
Moderate	⊕⊕⊕O
Low	⊕⊕OO
Very Low	⊕OOO

We downgraded the quality of evidence in all outcomes for risk of bias due to the lack of participant blinding, and in quality of life for the MCS for lack of assessor blinding. Inconsistency was downgraded in most cases due to heterogeneity in the results across studies, and is reflected in the I^2 statistics for each outcome. Indirectness was low in most outcomes, but downgraded for the change in anaerobic threshold as this was not a primary endpoint for most included studies, and was calculated indirectly from the measurement of $\dot{V}O_2$. Imprecision was graded as moderate to high due to study population numbers and our need to calculate means and standard deviations from other reported summary statistics. Publication bias was graded as low for most outcomes, except for the mental component of the quality of life score. This was downgraded due to the limited number of studies measuring this, and their relatively small sample sizes

© 2023 Clifford K et al. *JAMA Network Open*.

eTable 4. i-CONTENT Results for Included Studies' Therapeutic Quality

Study	Patient Selection	Dosage	Type of exercise programme	Qualified supervisor	Timing of outcome assessment	Safety of exercise program	Adherence to exercise program
Banerjee [35]	low risk	low risk	low risk	low risk	low risk	low risk	low risk
Barberan-Garcia [5]	low risk	low risk	low risk	low risk	low risk	low risk	low risk
Berkel [36]	low risk	low risk	low risk	low risk	low risk	low risk	low risk
Dunne [17]	low risk	low risk	low risk	high risk	low risk	low risk	low risk
Licker [37]	low risk	low risk	low risk	low risk	low risk	low risk	low risk
Molenaar [40]	low risk	low risk	low risk	high risk	low risk	low risk	low risk
Morkane [41]	low risk	low risk	low risk	high risk	low risk	low risk	low risk
Sebio-Garcia [43]	low risk	low risk	low risk	low risk	low risk	low risk	low risk
Stefanelli [39]	low risk	low risk	low risk	high risk	low risk	low risk	low risk
Van Rooijen [42]	low risk	low risk	low risk	high risk	low risk	low risk	low risk
West [16]	low risk	low risk	low risk	high risk	low risk	low risk	low risk
Woodfield [6]	low risk	low risk	low risk	low risk	low risk	low risk	low risk

© 2023 Clifford K et al. *JAMA Network Open*.

Funnel plots

Our analysis found evidence of studies outside the 95% psuedo confidence limits of the funnel plot for different endpoints, falling into a range of patterns. Different patterns were present for differnet enpoints in the same paper.

This included:

a) Studies outside the funnel plot showing changes less than the overall mean difference. This was the case for $\dot{V}O_{2peak}$ and LOS.
b) Studies outside the funnel plot showing changes greater than the overall mean difference (as may occur with publication bias). This was the case for 6 MWT and PPO.
c) Outlying studies on both sides of the funnel plot. This was the case for AT and MCS.

There were no studies outside the 95% limits of the funnel plot for the number of patients with complications or the physical component scores of the quality of life questionnaire.

The funnel plot for $\dot{V}O_{2peak}$ shows most studies have a similar standard error and report positive mean differences (favouring HIIT) with a significant effect. This indicates that the asymmetry in the funnel plot may be due to factors other than small study bias or selection bias. Augmenting the analysis to improve the symmetry of the funnel plot[1] would suggest that that there may be five missing studies (Figure S2), and imputing estimated results from these confirms the results of our pooled analysis. Tf(4.23 (2.7, 5.8), p<0.001). The imputed studies would have an higher estimated mean difference which is significant (p <0.01). There are two reasons why this doesn't support publication bias. Firstly the greater difference (greater benefit of the intervention) would be highly publishable, and secondly the larger improvements in $\dot{V}O_{2peak}$ are biologically implausible. We believe the asymmetry is likely to be related to the heterogeneity of study protocols rather than publication bias.

The broad range of distribution of endpoint results outside the funnel plots – with those that may fit publication bias being more 'minor' or secondary physiological endpoints, some endpoints having results on both sides of the funnel plot, and a some clinical endpoints all falling inside the funnel plot may also fit with heterogeneity rather than publication bias as the main contributing cause.

© 2023 Clifford K et al. *JAMA Network Open*.

eFigure 2. Funnel Plot for Meta-analysis of Change in $\dot{V}O_{2peak}$ in Included Studies

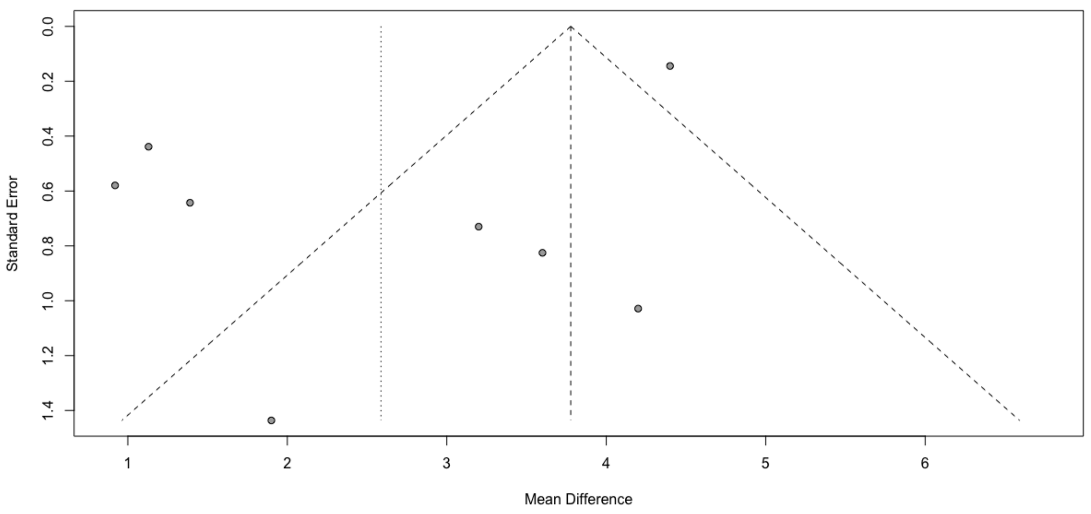

eFigure 3. Funnel Plot for Pooled Analysis of Studies Comparing HIIT With Standard Care on $\dot{V}O_{2peak}$ With Included Missing Studies

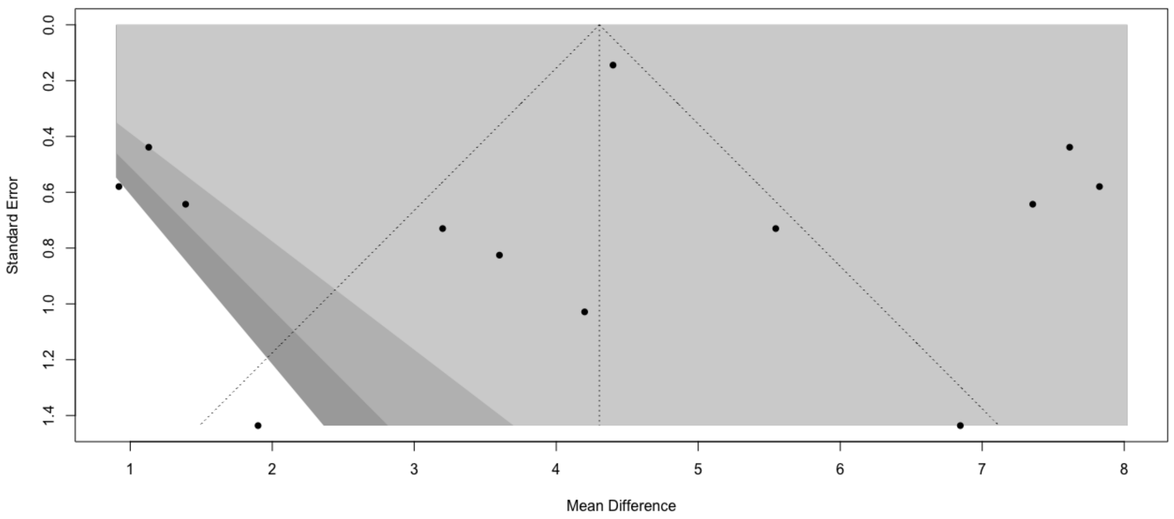

© 2023 Clifford K et al. *JAMA Network Open*.

Multi-vs Uni Modal prehabilitation

eFigure 4. Forest Plot for Change in $\dot{V}O_{2peak}$ Subgrouped by Number of Prehabilitation Interventions Included in Individual Studies

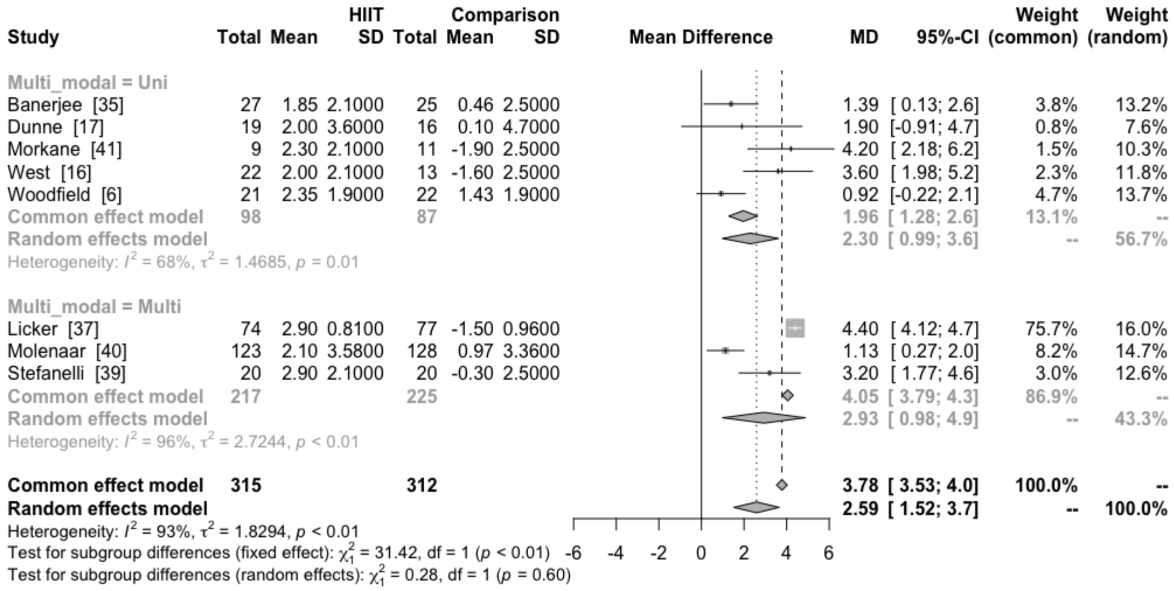

Three studies documenting the change in $\dot{V}O_{2peak}$ utilised multimodal prehabilitation programs (HIIT + Resistance + another intervention), and five studies utilised HIIT only prehabilitation (Figure S10). Random effects analysis shows that the change in $\dot{V}O_{2peak}$ did not differ between studies using multimodal prehabilitation and those using uni-modal prehabilitation (2.30 vs 2.93 mL·kg^{-1}·min^{-1} respectively) (p=0.60). The common effect model showed significant differences, however we cannot claim differences in the pooled analysis due to the wide confidence intervals and heterogenious individual study results.

eFigure 5. Forest Plot Showing the Mean Difference Between HIIT and Control Groups, Stratified by RCT and Cohort Study Results

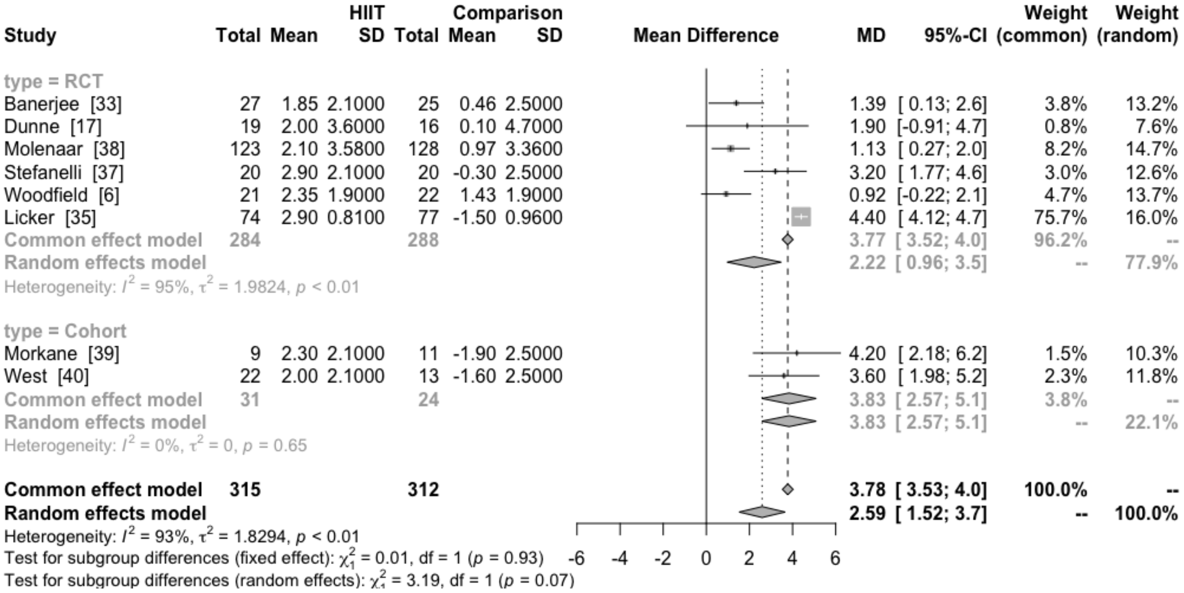

© 2023 Clifford K et al. *JAMA Network Open*.

eFigure 6. Funnel Plot for Meta-analysis of Change in 6-Minute Walk Test in Included Studies

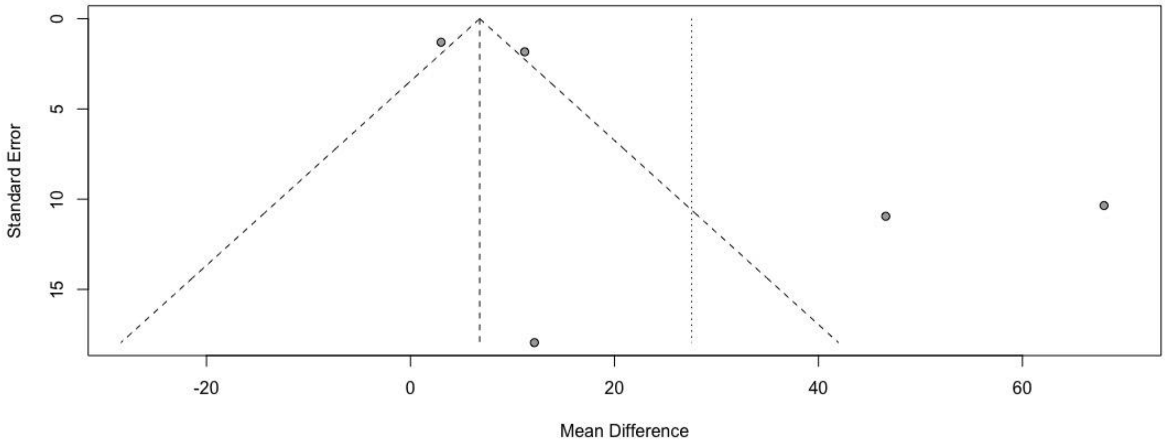

eFigure 7. Funnel Plot for Meta-analysis of Change in Peak Power Output in Included Studies

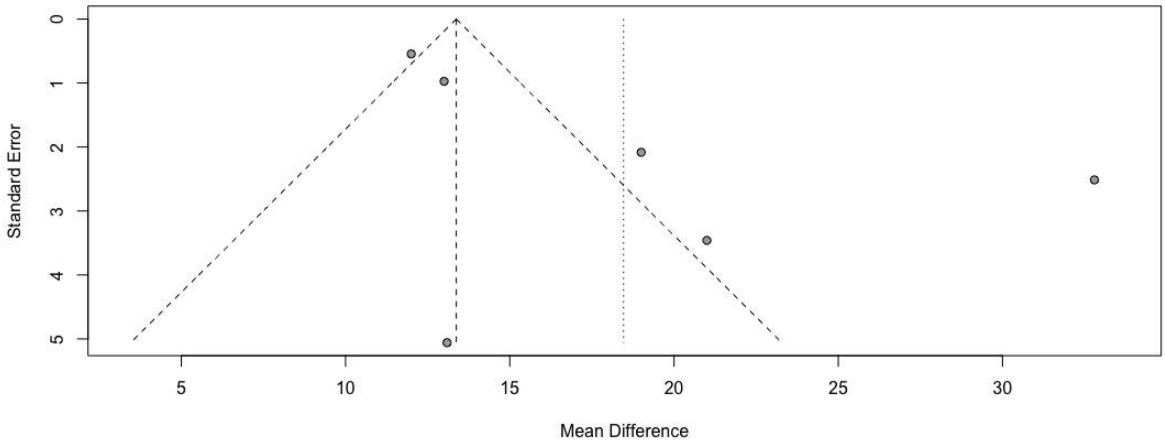

eFigure 8. Funnel Plot for Meta-analysis of Change in Anaerobic Threshold in Included Studies

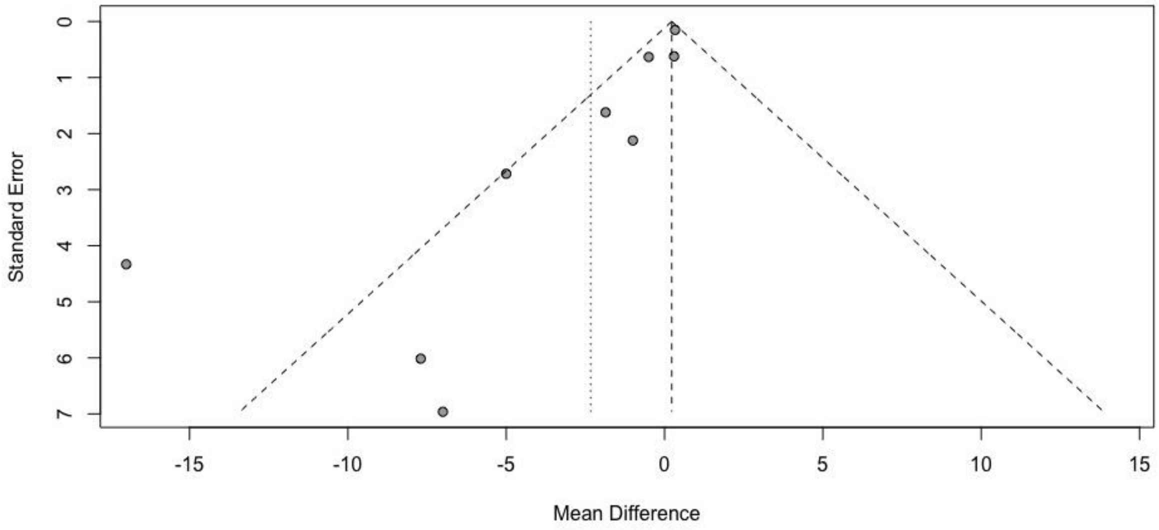

© 2023 Clifford K et al. *JAMA Network Open*.

eFigure 9. Funnel Plot for Meta-analysis of Number of Patients With Complications in Included Studies

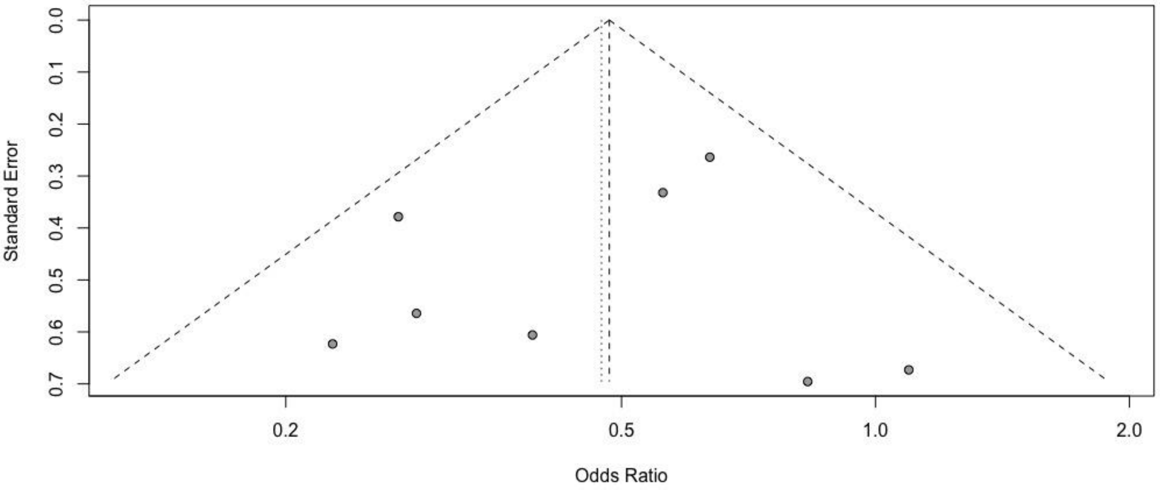

eFigure 10. Forest Plot for Number of Patients With Complications Subgrouped by Number of Prehabilitation Interventions Included in Individual Studies

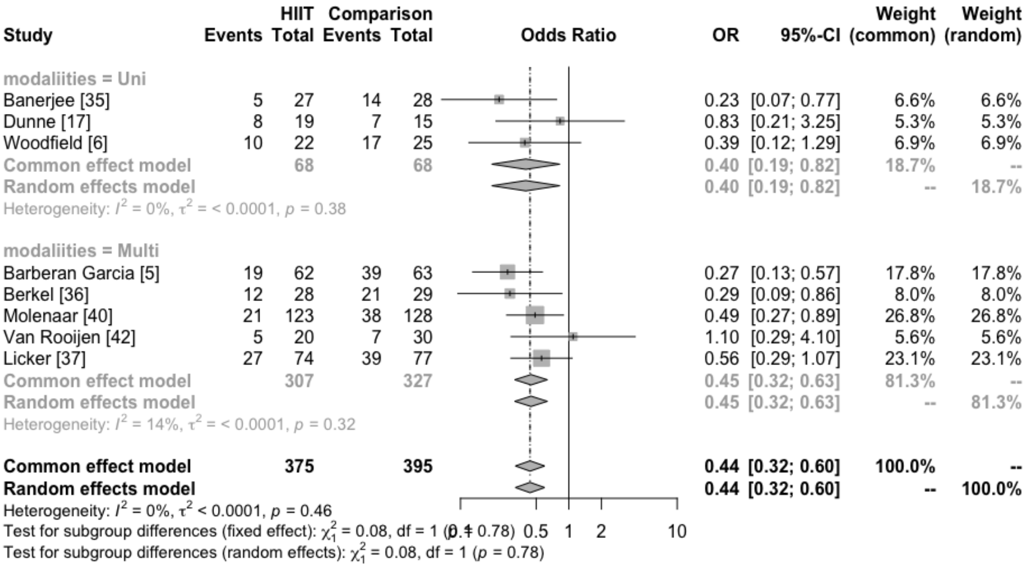

Five studies utilised multimodal prehabilitation programs (three combining HIIT with motivational counselling and three combining HIIT and resistance training) and three studies utilised HIIT only prehabilitation in the comparison on complication rates (Figure S11). Random effects analysis shows that the number of patients with post-operative complications did not differ between studies using multi- or uni-modal prehabilitation (OR 0.4 vs 0.43 respectively (p=0.87)).

© 2023 Clifford K et al. *JAMA Network Open*.

eFigure 11. Funnel Plot for Meta-analysis of Length of Stay in Included Studies

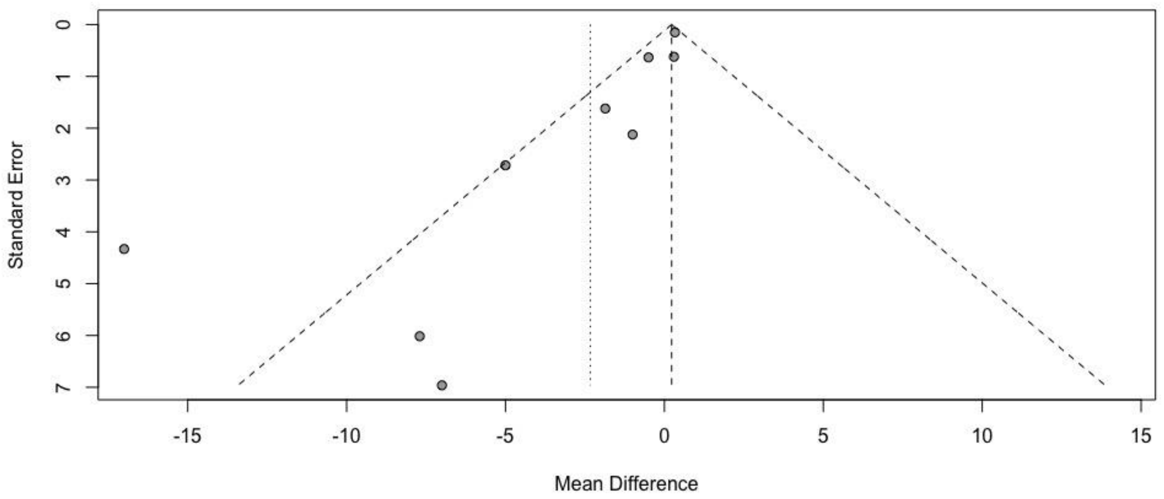

eFigure 12. Funnel Plot for Change in Physical Component Scores of the SF-36 Quality of Life Questionnaire

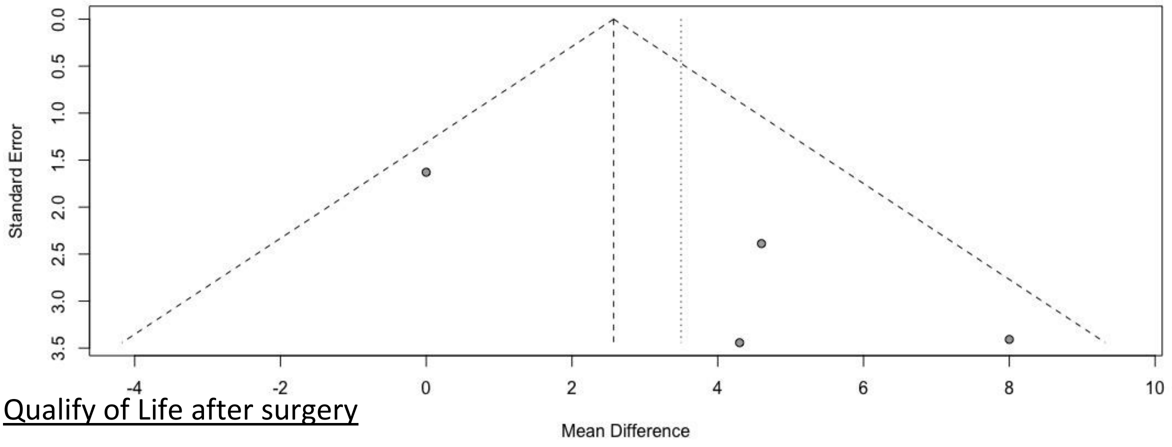

Qualify of Life after surgery

eFigure 13. Pooled Analysis for Studies Assessing the Physical Component Score of the SF-36 Quality of Life Questionnaire at Baseline and at 3 Months Postsurgery (Sebio-Garcia et al) and 12 Weeks (Woodfield et al)

Study	HIIT Total	HIIT Mean	HIIT SD	Comparison Total	Comparison Mean	Comparison SD	MD	95%-CI	Weight (common)	Weight (random)
Sebio Garcia 2016	9	4.30	4.0000	10	-4.80	5.8000	9.10	[4.66; 13.54]	72.0%	58.3%
Woodfield 2022	22	0.72	12.7400	25	-1.39	12.1200	2.11	[-5.03; 9.24]	28.0%	41.7%
**Common effect model**	**31**			**35**			**7.15**	**[3.37; 10.92]**	**100.0%**	**--**
**Random effects model**							**6.18**	**[-0.58; 12.94]**	**--**	**100.0%**

Heterogeneity: $I^2 = 62\%$, $\tau^2 = 15.2604$, $p = 0.10$

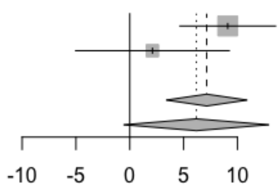

© 2023 Clifford K et al. *JAMA Network Open*.
