# Supplementary material for: Association of Preoperative High-Intensity Interval Training With Cardiorespiratory Fitness and Postoperative Outcomes Among Adults Undergoing Major Surgery: A Systematic Review and Meta-Analysis
Source: JAMA Netw Open. 2023 Jun 30;6(6):e2320527. doi: 10.1001/jamanetworkopen.2023.20527 (PMC10314310; doi:10.1001/jamanetworkopen.2023.20527)
Supplement: Supplement 2. — Data Sharing Statement [file jamanetwopen-e2320527-s002.pdf]

Data Sharing Statement

Clifford. Association of Preoperative High-Intensity Interval Training With Cardiorespiratory Fitness and Postoperative Outcomes Among Adults Undergoing Major Surgery. *JAMA Netw Open*. Published June 30, 2023. doi:10.1001/jamanetworkopen.2023.20527

Data
Data available: Yes
Data types: Deidentified participant data
How to access data: upon request to the corresponding author
When available: With publication

Supporting Documents
Document types: None

Additional Information
Who can access the data: researchers whose proposed use of the data has been approved
Types of analyses: for any purpose
Mechanisms of data availability: after approval of a proposal
Any additional restrictions: none
